# Novel Internet Support for Neck-Specific Rehabilitation Improves Work-Related Outcomes to the Same Extent as Extensive Visits to a Physiotherapy Clinic in Individuals with Chronic Whiplash-Associated Disorders: A Prospective Randomised Study

**DOI:** 10.1007/s10926-024-10176-0

**Published:** 2024-03-25

**Authors:** Anneli Peolsson, Emma Nilsing Strid, Gunnel Peterson

**Affiliations:** 1 Occupational and Environmental Medicine Centre, Department of Health, Medicine and Caring Sciences, Unit of Clinical Medicine, Linköping University, 58185 Linköping, Sweden; 2 Department of Health, Medicine and Caring Sciences, Unit of Physiotherapy, Linköping University, Linköping, Sweden; 3 Faculty of Medicine and Health, University Health Care Research Centre, Örebro University, Örebro, Sweden; 4 Centre for Clinical Research Sörmland, Uppsala University, Uppsala, Sweden

**Keywords:** Whiplash Injuries, Exercise Therapy, Internet-Based Intervention, Spine, Work

## Abstract

**Purpose:**

To address the current lack of information about work-related factors for individuals with whiplash-associated disorders (WAD) we investigated the effectiveness of 3 months of neck-specific rehabilitation with internet support in combination with four physiotherapy visits (NSEIT) compared to the same exercises performed twice a week (24 times) at a physiotherapy clinic (NSE).

**Methods:**

This is a prospective, multicentre, randomised controlled trial regarding secondary outcomes of work-related factors in 140 individuals with chronic moderate/severe WAD with 3- and 15-month follow-up.

**Results:**

There were no group differences between NSE and NSEIT in the Work Ability Scale or work subscales of the Neck Disability Index, Whiplash Disability Questionnaire or Fear Avoidance Beliefs Questionnaire (FABQ-work). Both groups improved in all work-related outcome measures, except for FABQ-work after the 3-month intervention and results were maintained at the 15-month follow-up. Conclusions: Despite fewer physiotherapy visits for the NSEIT group, there were no group differences between NSEIT and NSE, with improvements in most work-related measures maintained at the 15-month follow-up. The results of the present study are promising for those with remaining work ability problems after a whiplash injury.

Protocol registered before data collection started: clinicaltrials.gov NCT03022812.

## Introduction

Whiplash-associated disorders (WAD) after e.g., a car crash is common, with a cumulative annual incidence as high as 600 per 100,000 inhabitants [1]. About half of car crash victims affected by a whiplash trauma experience chronic and disabling WAD [2, 3], with frequent and intense pain, sleeping problems, and reduced health and societal participation. A high cost following WAD is frequently reported [3], mainly due to reduced ability to work compared to before the car crash. Those with WAD are less likely to return to work than those with other musculoskeletal injuries [4] and reduced ability to work is a commonly identified problem in individuals with WAD [5]. It has been recommended that work functioning should be part of a core outcome set for clinical trials involving WAD [6], something that has scarcely been evaluated in chronic moderate/ severe WAD after an exercise intervention [7, 8]. Being able to work has been shown to be important for general health [9–11], but work ability is also important for maintaining treatment gains in physical and psychological outcome measures [12], is thus of utmost importance to investigate after treatment.

The treatment evidence for WAD is inconclusive [13–15]. However, neck-specific exercises for those with moderate/severe chronic disabling WAD have seldom been evaluated [15, 16]. In a randomised controlled trial (RCT) of individuals with moderate/severe chronic WAD, neck-specific exercises have shown good and superior results to general physical activity regarding pain, function and work ability [7, 17]. However, the number of physiotherapy visits was extensive (twice a week for three months; 24 visits altogether). More efficient, flexible ways of delivering neck-specific exercises with fewer visits are needed, especially for working individuals. E-health solutions have previously been shown to be effective for other musculoskeletal conditions [18–21]. Recently, the main results of our RCT regarding internet support for rehabilitation in chronic WAD was published [22] showing improvements over time regarding pain and disability. However, work-related factors were not presented.

The aim of this prospective, multicentre RCT was to compare the effects of an Internet-based, neck-specific rehabilitation programme in combination with four physiotherapy visits (NSEIT) to the same rehabilitation programme performed at a physiotherapy clinic (NSE) twice a week for three months (24 visits) regarding the work-related secondary outcomes of the RCT. The hypothesis was that there should be no group differences and that both groups will improve.

## Material and Methods

### Design

This is a prospective, multicentre, randomised controlled trial [22, 23] with investigators blinded for randomisation. The trial interventions were delivered by 57 physiotherapists in a primary healthcare setting or working in private outpatient practice in ten county councils in Sweden. It compares two different ways of distributing neck-specific rehabilitation programme: NSEIT or NSE to 140 individuals with chronic WAD. Measurements were made at baseline, 3 months (end of treatment), and 15 months (12 months after the end of the intervention) and include ratings of work-related factors. The study was approved by the regional ethics committee in Linköping, Sweden (2016/135-31) [23] (ClinicalTrials.gov; NCT03022812). Data were collected between 6 April 2017 and 15 September 2020.

### Randomisation

A computerized block randomisation list stratified by gender was used for randomisation into two groups, NSEIT or NSE, with 70 individuals per group [22, 23].

### Study Criteria for Participation

Information about the study was made available by healthcare providers and through advertising in newspapers, on posters, social media, and the university’s website. Interested individuals contacted the research team through the website. Study criteria was ensured through several steps: brief survey, telephone interview and physical examination [22, 23] before inclusion. Baseline measurements were completed before inclusion [22, 23].

Inclusion criteria [22, 23]: Individuals with a whiplash injury from a traffic accident involving a four-wheeled motor vehicle at least 6 months but less than 5 years previously with chronic neck problems corresponding to WAD grades II–III [24] verified by clinical examination. Additional inclusion criteria were: neck symptoms within the first week after the injury, average estimated pain in the previous week of at least 20 mm on the visual analogue scale (VAS) [25], neck disability of more than 20% on the Neck Disability Index (NDI) [26], being of working age (18–63 years), having daily access to a computer/tablet/smartphone and the Internet and sufficient self-estimated time to follow the treatment programme [22, 23].

Exclusion criteria [22, 23]: Individuals with signs of head injury at the time of whiplash injury were excluded. Additional exclusion criteria were: previous fractures or dislocation of the cervical column; known or suspected serious physical pathology; previous severe neck problems that resulted in sick leave of more than 1 month during the year before the current whiplash injury; surgery in the cervical column; generalised or more dominant pain elsewhere in the body; other illness/injury that may prevent full participation in the study; inability to understand and write in Swedish; diagnosed severe mental illness, such as psychosis, schizophrenia, or personality disorders; current alcohol or drug abuse [22, 23]; participation in neck-specific exercises in an earlier research study [7, 17].

### Participants

**Fig. 1:**
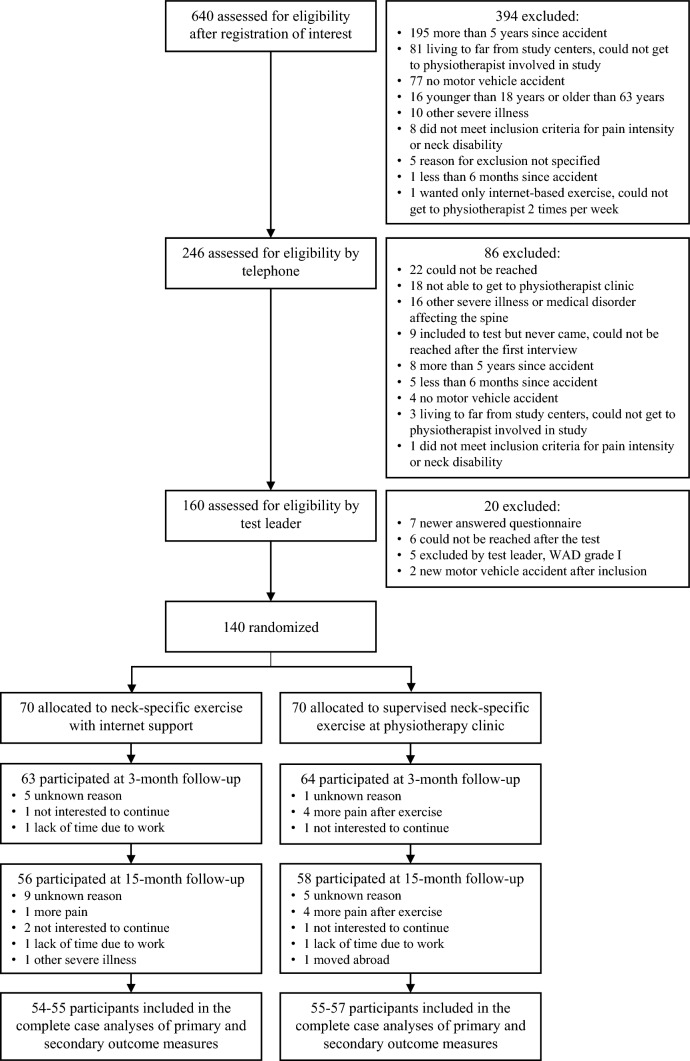
The consort flow diagram

A total of 140 participants, 70 in each group, participated in the study (Fig. 1). They had a mean age of 40.5 years (SD ± 11.5), 79% were women, and they had a mean duration of WAD of 26 months (SD ± 16.8) (Table 1). Most participants were full-time or part-time workers (81%) or students (11%) (Table 1).

**Table 1:** Baseline characteristics of participants with whiplash-associated disorders (WAD) grade II and III, by treatment group

	NSEIT (n = 70)	NSE (n = 70)
Age, mean ± SD	40.4 ± 11.6	40.5 ± 11.4
Sex, female, n (%)	55 (79%)	55 (79%)
Months since injury, mean ± SD	27.4 ± 21.0	25.2 ± 15.5
WAD grade, grade II, n (%)	46 (66%)	43 (61%)
Educational level, n (%)		
Elementary	1 (1%)	0 (0%)
High school	30 (43%)	39 (57%)
University	35 (50%)	27 (39%)
Other	4 (6%)	3 (4%)
Marital status, married/cohabiting, n (%)	52 (74%)	53 (76%)
Previous treatment, yes, n (%)	62 (89%)	66 (94%)
Use of analgesic drugs, yes, n (%)	57 (81%)	60 (86%)
Reported to insurance company, n (%)		
No	12 (17%)	10 (14%)
Yes, not approved	10 (14%)	5 (7%)
Yes, under investigation	19 (27%)	13 (19%)
Yes, approved	29 (41%)	42 (60%)
Occupation, n (%)		
Armed forces occupations	0 (0%)	0 (0%)
Managers	4 (6%)	7 (10%)
Occupations requiring advanced level of higher education	20 (29%)	13 (19%)
Occupations requiring higher education qualifications or equivalent	5 (7%)	12 (17%)
Administration and customer service clerks	6 (9%)	11 (16%)
Service, care, and shop sales workers	19 (28%)	14 (20%)
Agricultural, horticultural, forestry, and fishery workers	3 (4%)	1 (1%)
Building and manufacturing workers	1 (1%)	3 (4%)
Mechanical manufacturing and transport workers, etc	4 (6%)	1 (1%)
Elementary occupations	0 (0%)	2 (3%)
Students	6 (9%)	6 (9%)
Unemployed/Jobseekers	1 (1%)	0 (0%)
Sick-leave, n (%)		
No	61 (87%)	62 (89%)
Sick-leave, part-time	6 (9%)	5 (7%)
Sick-leave, full-time	3 (4%)	3 (4%)
Stanford Presenteeism Scale 6*, Total score (6–30), mean ± SD Total score interval, n (%)	22.1 ± 4.6	22.4 ± 4.6
6–10 Low presenteeism	0 (0%)	0 (0%)
11–15	4 (7%)	4 (7%)
16–20	20 (34%)	15 (26%)
21–25	16 (28%)	22 (39%)
26–30 High presenteeism	18 (31%)	16 (28%)
Effort Reward Imbalance	0.74 ± 0.22	0.75 ± 0.26
Disability, mean ± SD		
Neck Disability Index (0–100%)	39.4 ± 12.2	36.6 ± 10.8
Whiplash Disability Questionnaire (0–130)	56.7 ± 21.7	56.8 ± 22.4
Pain intensity, mean ± SD		
Neck pain intensity now, VAS (0–100 mm)	34.6 ± 21.4	39.7 ± 22.4
Arm pain intensity now, VAS (0–100 mm)	16.7 ± 21.0	15.5 ± 21.5
Health-related quality of life, mean ± SD		
EQ-5D-3L index (−0.594 to 1)	0.57 ± 0.29	0.64 ± 0.21
EQ thermometer / VAS (0–100 mm)	57.7 ± 18.7	58.6 ± 17.0
Work-related outcome factors, mean ± SD		
Work Ability Score, NRS (0–10)	6.4 ± 2.4	6.6 ± 1.8
Neck Disability Index—Work, (0–5)	1.8 ± 1.1	1.6 ± 1.0
Whiplash Disability Questionnaire—Impact on work, NRS (0–10)	5.0 ± 2.5	4.7 ± 2.2
Fear Avoidance Beliefs Questionnaire—Work, (0–42)	13.1 ± 9.5	13.4 ± 9.9

*EQ-5D-3L* EuroQol 5 dimensions with 3 level answers; *NRS* Numeric Rating Scale; *NSEIT* Neck-specific exercises with internet support; *NSE* Neck-specific exercises at physiotherapy clinic; *SD* Standard deviation; *VAS* Visual Analog Scale, *WAD* Whiplash-associated disorders

^*^Stanford Presenteeism Scale was only answered by participants working or studying with pain (n = 116)

### Interventions

Both groups performed neck-specific rehabilitation programme for 12 weeks: NSEIT (Internet-based support exercises in combination with four visits to the physiotherapist) or NSE (exercises at a physiotherapy clinic twice/week) [22, 23]. Details of the rehabilitation programme and the education of physiotherapists are described elsewhere [22, 23]. The same physiotherapist could do both interventions.

#### NSEIT

The participants underwent a 12-week neck-specific rehabilitation programme that included only four visits to the physiotherapist. The exercises were performed and most of the information was given with the help of a non-interactive Internet support outside the healthcare system [22, 23]. With minor adjustments, these exercises had been used with good results in a previous RCT [7, 17] (DOI https://doi.org/10.3384/report.diva-113,865).

#### NSE

In the NSE group, participants received the same information and neck-specific rehabilitation program as the NSEIT group but delivered by a physiotherapist [22, 23]. The participants had a total of 24 visits with the physiotherapist (twice a week for 12 weeks). The individuals also performed the exercises at home.

### Outcome Measures

The collected background data included: age; sex; marital status; WAD grade; previous treatment for WAD; symptom duration; educational level; occupational classification (“Standard for Swedish work classification” SSY code [27]); NDI [26]; Whiplash Disability Questionnaire (WDQ) [28–30]; pain intensity [25]; self-assessment of sick leave; sickness presence (working despite pain; Stanford presenteeism scale) [31]; and Fear-Avoidance Beliefs Questionnaire (FABQ) [32, 33] (Table 1).

Primary outcome measure in the present paper: The Work Ability Score (WAS) [34], also named the single item (question number 1 in the Work Ability Index). In the WAS, present work ability is compared to lifetime best. It is well validated and has a scoring range of 0–10 points, with higher scores indicating greater work ability [34].

Secondary outcome measures in the present paper: neck-related function as measured by the NDI (measures the degree of perceived pain or disability status based on daily activities and underlying neck pain) question 7 regarding work (0 = I can do as much work as I want to/ no disability, 5 = I cannot do any work at all/ worst possible disability) [26] and WDQ (provide information on the impact that the WAD has upon lifestyle) question 3 regarding impact on work “Do your whiplash symptoms interfere with your work/home/study duties?” (0 = not at all/ no disability, 10 = unable to perform/worst possible disability) [28–30]. FABQ-work investigates fear-avoidance beliefs in a clinical setting and is the second of two FABQ subscales (work items 6–16, but only summarizing the score of items 6, 7, 9, 10, 11, 12, and 15) as follows: My pain was caused by my work or by an accident at work; My work aggravates my pain; My work is too heavy for me; My work makes or would make my pain worse; My work might harm my neck; I should not do my normal work with my present pain; I do not think that I will be back to my normal work within three months (0 = completely disagree, 6 = completely agree, with a maximum of 42 points indicating severe fear-avoidance beliefs) [32, 33].

### Statistical Analysis

All statistical analyses were performed using IBM SPSS Statistics for Windows (Version 28.0. Armonk, NY: IBM Corp). Participants’ characteristics, background data, and estimates of outcome measures at baseline are presented for NSEIT and NSE in Table 1. Frequencies and percentages are presented for binary and other categorical variables and means with standard deviations for all numerical discrete and continuous variables.

A mixed design ANOVA (3 time points × 2 treatment groups) was used to analyse within-group changes between time points (baseline, 3 months, and 15 months follow-up) and between-group differences at each time point, as well as changes between time points in all work-related outcome factors: WAS, NDI work (Sect. 7), WDQ Impact on work (question 3), and FABQ-work. The mixed design ANOVA was used under the assumption that outcome data was multivariate and normally distributed, and that any missing data was missing at random. Mauchly’s test of sphericity was used to test the assumption of sphericity in repeated measures. If this assumption was violated (*p* < 0.05), the Greenhouse–Geisser corrected epsilon was used. The Bonferroni correction was used for all pairwise contrasts: change baseline to 3 months, change baseline to 15 months, and change 3 to 15 months. All effects were reported as significant at *p* < 0.05. Partial eta squared (*η*_*p*_^*2*^) was reported as a measure of effect size; 0.01 indicates a small effect, 0.06 indicates an intermediate effect, and 0.14 indicates a large effect [35]. The results of the mixed design ANOVAs are presented in Table 2.

**Table 2:** Mixed Design ANOVA (3 time points × 2 treatment groups) on work-related outcome factors of participants with whiplash-associated disorders (WAD) grades II and III

Work-related outcome factors	Time point		NSEIT (*n* = 55)	NSE (*n* = 57)	Between-group difference (NSEIT–NSE)
			Mean (95% CI);* p-value*	Mean (95% CI);* p-value*	Mean (95% CI);* p-value*
Work Ability Score, NRS (0–10) *“How many points would you give your current work ability?” (0* = *cannot work at all; 10* = *work ability at its best)*	Baseline ^(1)^		6.5 (5.9 to 7.1)	6.5 (6.0 to 7.1)	−0.1 (-0.9 to 0.8); *p* = *0.835*
	3 months ^(2)^		7.2 (6.7 to 7.8)	7.3 (6.8 to 7.8)	−0.0 (−0.8 to 0.7); *p* = *0.903*
	15 months ^(3)^		7.1 (6.6 to 7.6)	7.6 (7.0 to 8.1)	−0.5 (−1.2 to 0.3); *p* = *0.214*
	Within-group change	(1)–(2)	−0.8 (−1.3 to −0.2); *p* = *0.003*	−0.7 (−1.3 to −0.2); *p* = *0.004*	−0.0 (−0.7 to 0.6); *p* = *0.889*
		(1)–(3)	−0.6 (−1.3 to −0.0); *p* = *0.040*	−1.0 (−1.6 to −0.4); *p* < *0.001*	0.4 (−0.3 to 1.1); *p* = *0.285*
		(2)–(3)	0.1 (−0.3 to 0.6); *p* = *1*	−0.3 (−0.7 to 0.2); *p* = *0.443*	0.4 (−0.1 to 1.0); *p* = *0.971*
Neck Disability Index*—*Sect. 7, Work, (0–5)	Baseline ^(1)^		1.8 (1.5 to 2.1)	1.7 (1.4 to 2.0)	0.1 (−0.3 to 0.5); *p* = *0.570*
	3 months ^(2)^		1.2 (0.9 to 1.5)	1.3 (1.0 to 1.6)	−0.0 (−0.4 to 0.4); *p* = *0.898*
	15 months ^(3)^		1.2 (0.9 to 1.5)	1.0 (0.8 to 1.3)	0.2 (−0.2 to 0.6); *p* = *0.327*
	Within-group change	(1)–(2)	0.6 (0.3 to 0.8); *p* < *0.001*	0.4 (0.2 to 0.7); *p* < *0.001*	0.1 (−0.2 to 0.4); *p* = *0.337*
		(1)–(3)	0.6 (0.3 to 0.9); *p* < *0.001*	0.7 (0.3 to 1.0); *p* < *0.001*	−0.1 (−0.4 to 0.3); *p* = *0.727*
		(2)–(3)	0.0 (−0.3 to 0.3); *p* = *1*	0.2 (−0.0 to 0.5); *p* = *0.116*	−0.2 (−0.5 to 0.1); *p* = *0.214*
Whiplash Disability Questionnaire*—*Impact on work, NRS (0–10) *“Do your whiplash symptoms interfere with your work/ home/ study duties?” (0* = *not at all; 10* = *unable to perform)*	Baseline ^(1)^		4.9 (4.2 to 5.6)	4.6 (4.0 to 5.3)	0.3 (−0.7 to 1.2); *p* = *0.557*
	3 months ^(2)^		3.3 (2.7 to 3.9)	3.4 (2.8 to 4.0)	−0.1 (−0.9 to 0.8); *p* = *0.894*
	15 months ^(3)^		3.3 (2.6 to 3.9)	3.0(2.4 to 3.6)	0.3 (−0.7 to 1.2); *p* = *0.587*
	Within-group change	(1)–(2)	1.6 (0.9 to 2.3); *p* < *0.001*	1.3 (0.6 to 2.0); *p* < *0.001*	0.3 (−0.5 to 1.2); *p* = *0.422*
		(1)–(3)	1.7 (1.0 to 2.3); *p* < *0.001*	1.6 (1.0 to 2.3); *p* < *0.001*	0.0 (−0.7 to 0.8); *p* = *0.953*
		(2)–(3)	0.1 (−0.6 to 0.7); *p* = *1*	0.4 (−0.2 to 1.0); *p* = *0.409*	−0.3 (−1.0 to 0.4); *p* = *0.372*
Fear Avoidance Beliefs Questionnaire—Work, (0–42)	Baseline ^(1)^		13.5 (10.7 to 16.2)	13.9 (11.2 to 16.6)	−0.4 (−4.3 to 3.4); *p* = *0.827*
	3 months ^(2)^		12.0 (9.3 to 14.8)	12.7 (10.0 to 15.4)	−0.6 (−4.5 to 3.2); *p* = *0.744*
	15 months ^(3)^		10.9 (8.2 to 13.5)	11.9 (9.3 to 14.6)	−1.1 (−4.8 to 2.6); *p* = *0.562*
	Within-group change	(1)–(2)	1.4 (−1.2 to 4.1); *p* = *0.542*	1.2 (−1.3 to 3.8); *p* = *0.741*	0.2 (−2.8 to 3.2); *p* = *0.891*
		(1)–(3)	2.6 (−0.0 to 5.3); *p* = *0.054*	2.0 (−0.7 to 4.6); *p* = *0.218*	0.7 (−2.4 to 3.7); *p* = *0.666*
		(2)–(3)	1.2 (−1.4 to 3.7); *p* = *0.782*	0.7 (−1.8 to 3.3); *p* = *1*	0.5 (−2.5 to 3.4); *p* = *0.757*

*CI* Confidence interval; *NRS* Numeric Rating Scale; *NSEIT* Neck-specific exercises with internet support; *NSE* Neck-specific exercises at physiotherapy clinic; *SD* Standard deviation; *WAD* Whiplash-associated disorders

We performed a response analysis based on complete responders in WAS at all three time points; complete responders (NSEIT, *n* = 55; NSE, *n* = 57) versus non-complete responders (NSEIT, *n* = 15; NSE, *n* = 13). There was a significant difference (*p* = 0.002) between complete responders and non-complete responders regarding age, with non-complete responders in both treatment groups being younger. However, age was not correlated to change in any of the work-related outcome factors (Pearson correlation coefficient between 0.02 and 0.11).

## Results

### Primary Outcome Measure

**Fig. 2:**
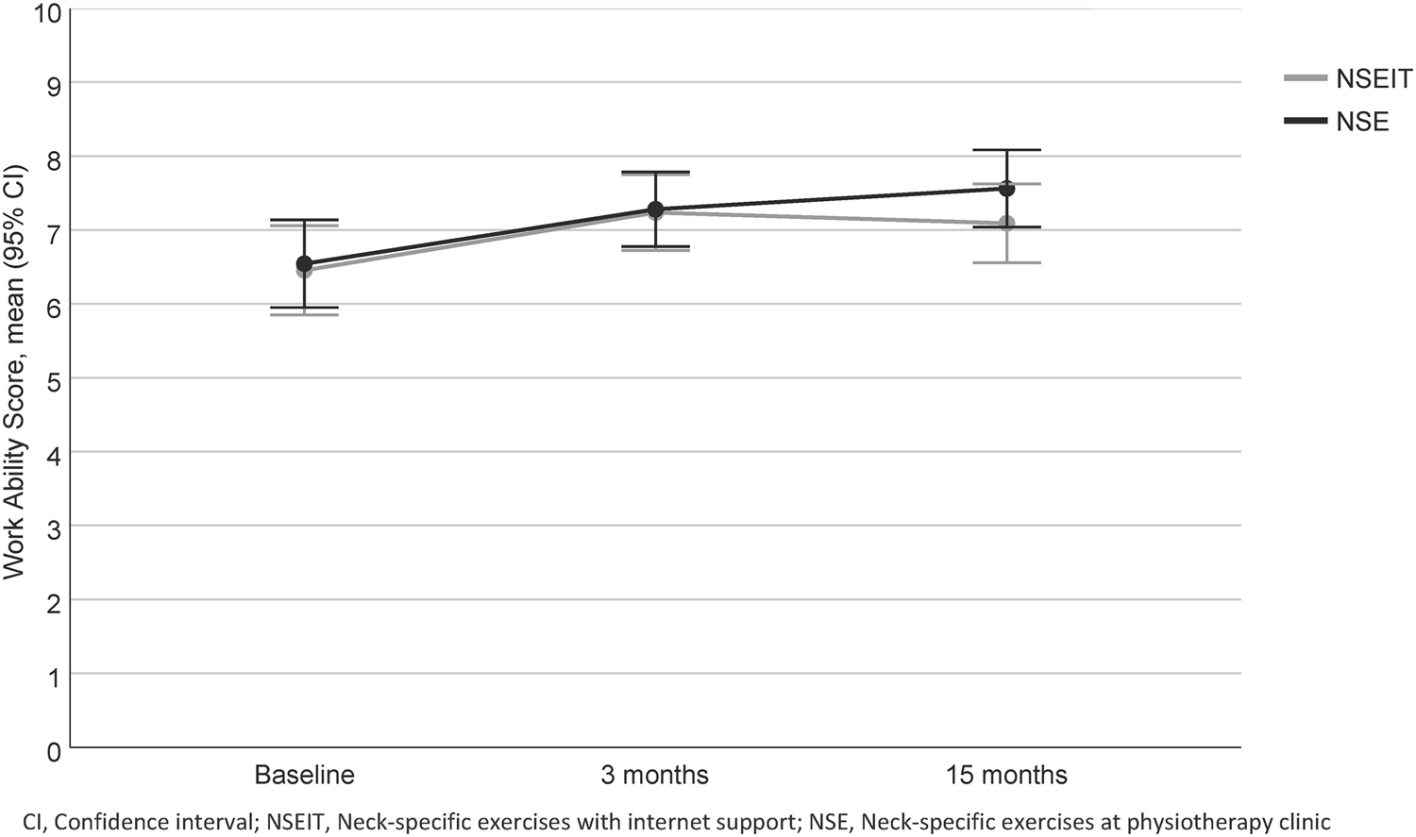
Outcome of Work-Ability Score (WAS) at baseline and at 3 and 15 month follow-up for both intervention groups

There was no significant interaction effect of time by treatment group of WAS (Table 2, Fig. 2). There was a significant main effect of WAS over time (whole group [*F(1.8, 220)* = *16.6, p* < *0.001, η*_*p*_^*2*^ = *0.13*]; NSEIT [*F(2, 109)* = *5.7, p* = *0.04, η*_*p*_^*2*^ = *0.10*], NSE [*F(2, 109)* = *8.5, p* < *0.001, η*_*p*_^*2*^ = *0.14*]). The pairwise contrasts showed significant within-group effects, between baseline and 3 months follow-up as well as between baseline and 15 months follow-up, in both NSEIT and NSE (Table 2, Fig. 2).

### Secondary Outcome Measures

There was no significant interaction effect of time by treatment group of NDI work (Table 2). There was a significant main effect of NDI work over time (whole group [*F(1.8, 202.9)* = *30.7, p* < *0.001, η*_*p*_^*2*^ = *0.22*]; NSEIT [*F(2, 109)* = *15.8, p* < *0.001, η*_*p*_^*2*^ = *0.23*], NSE [*F(2, 109)* = *8.5, p* < *0.001, η*_*p*_^*2*^ = *0.20*]). The pairwise contrasts showed significant within-group effects, between baseline and 3 months follow-up as well as between baseline and 15 months follow-up, in both NSEIT and NSE (Table 2).

There was no significant interaction effect of time by treatment group of WDQ work (Table 2). There was a significant main effect of WDQ impact on work over time (whole group [*F(2, 220)* = *42.8, p* < *0.001, η*_*p*_^*2*^ = *0.28*]; NSEIT [*F(2, 109)* = *19.9, p* < *0.001, η*_*p*_^*2*^ = *0.27*], NSE [*F(2, 109)* = *18.2, p* < *0.001, η*_*p*_^*2*^ = *0.25*]). The pairwise contrasts showed significant within-group effects, between baseline and 3 months follow-up as well as between baseline and 15 months follow-up, in both NSEIT and NSE (Table 2).

There was no significant interaction effect of time by treatment group of FABQ-work (Table 2). There was a significant main effect of FABQ-work over time for the whole group, but not when divided by treatment group (whole group [*F(2, 214)* = *4.7, p* = *0.01, η*_*p*_^*2*^ = *0.04*]; NSEIT [*F(2, 106)* = *2.9, p* = *0.06, η*_*p*_^*2*^ = *0.05*], NSE [*F(2, 109)* = *8.5, p* = *0.20, η*_*p*_^*2*^ = *0.03*]).

### Exercise Fulfilment

The participants’ self-reported compliance with exercise was higher in the NSE group (94%) than in the NSEIT group (75%, *p* = 0.006) during the 12-week intervention period.

## Discussion

The aim of the prospective, multicentre RCT was to compare the effects of NSEIT to NSE and in this article we describe the secondary outcomes of the RCT concerning work-related factors [22, 23]. Both groups improved in all work-related outcome measures, except for FABQ-work after the 3-month intervention, and results were maintained at the 15-month follow-up. This is confirming our hypothesis that NSEIT is as effective as NSE, but with fewer physiotherapy visits.

This is the first study [22, 23] investigating whether NSEIT is as effective as NSE, and in this paper we present the work-related outcomes. This makes the study unique and novel and is in line with the digitalization occurring throughout global society, responding to the need for increased flexibility, availability, patient independence, and reduced healthcare costs.

There is to our knowledge no quantitative study presenting the outcomes of Internet-delivered treatment investigating chronic WAD, severe WAD, and exercise for work-related factors in WAD. Bring et al. [18] reported that an Internet-delivered behavioural programme was as effective as clinic-based face-to-face therapy sessions in terms of psychosocial factors and that both interventions were superior to self-care instructions for individuals with mild/ moderate acute WAD. This is in line with the present results. The results are also in line with results from other studies showing E-health solutions to be effective for musculoskeletal conditions such as chronic back pain, knee osteoarthritis, total knee arthroplasty, rheumatoid arthritis, and vestibular rehabilitation, as well as other conditions [19, 21, 36–38], although work-related factors were not investigated in these studies.

According to Lelieveld et al. [39], an Internet-based programme should preferably be combined with a few visits to the caregiver. The authors strongly believe that the addition to the Internet-based programme of the four physiotherapy visits for an introduction, support, and to check that the exercises have been correctly performed was of great importance for the patient to feel safe, reassured, and motivated. However, this hypothesis needs to be investigated in future qualitative studies. The fact that both groups improved over time also confirms our earlier favourable results for NSE from two RCTs in which the same programme, although not an E-health solution, was used for chronic WAD patients [7, 17, 40]. Ferreira et al. [41] reported that freely accessible websites offering treatment recommendations for low back pain are mostly inaccurate, and this may also be the case for neck pain. Internet-based recommendations for treatment must be based on research, as in the present study.

As shown in our earlier RCT [7] where neck-specific exercise in combination with a behavioural approach had better outcome in work ability than only neck-specific exercises, the authors believe that the elements of behavioural components in the NSEIT/NSE in addition to the exercises were of importance to motivate and help the participants feel safe and reassured. Both groups improved over time and, except for FABQ-work, showed intermediate to large effect sizes. This is in contrast to the findings of a meta-analysis of exercise for the management of neck pain, including WAD, which showed significant improvements but small effect sizes [42]. As the present population has chronic moderate/severe WAD, which is generally recognized as difficult to treat with no spontaneous recovery to be expected after the first six months, these are especially promising results. In addition, the good results were maintained. Other interventions, such as ergonomic interventions, have not shown convincing results in preventing work disability [43].

In this study there was a small effect-size of improvement for FABQ-work, which may be explained by the fact that the participants already scored low values/low fear at baseline. This gave limited room for improvement, although they did close to significantly improve in the NSEIT group at 15-month follow-up.

Within-group changes of WAS were 0.6 to 1.0 from baseline to 15-month follow-up in NSEIT/NSE in the present study. For low back pain, improvements of 1.5 were interpreted as clinically important [44]. Clinically relevant improvement data is yet unavailable for WAS and the chronic WAD population and needs to be investigated in a future study. For this reason, we cannot comment on whether the improvement in the current study was clinically relevant or not.

Work ability measured with WAS, the main outcome in the present study, is a broader concept than work capacity, return to work or being on sick leave. Work ability has been variously defined, e.g., in the work ability house model [45], as the balance between work and individual resources. As the social insurance system differs between countries, work ability and not sick leave may be a preferred outcome. Although it is influenced by individual resources and working conditions, which need to be understood by healthcare professionals [8]. The results of a meta-analysis show that half of those with WAD who return to work display reduced work ability [46]. This may lead to presenteeism or to choose to remain outside the insurance medical system, being dependence to relatives. There are only a few studies, mostly cross-sectional, regarding ability to work in WAD patients, and they use different measures [11]. Apart from our previous RCT [7], showing neck-specific exercises to be superior to prescribed physical activity, there is to our knowledge no RCT of merely physical interventions in chronic WAD that investigates work ability as an outcome measure. This strengthens the importance and scientific contribution of the present study. Work is important across a broad range of aspects, including social roles, socialisation, context, status, and general health, and not only from an economic perspective [47, 48]. Thus, it is important to investigate work as an outcome after interventions [6, 9–11].

### Strengths and Weaknesses of the Study

A strength of the present study was that it is a randomised controlled multicentre trial including individuals with chronic moderate/severe WAD often being excluded in rehabilitation studies. Another strength is that the good results were not only observed after the 3-month intervention period but were also maintained at the 15-month follow-up. This confirms the results of the previously presented main outcome of pain and neck-specific disability [22]. Apart from the study by Bring et al. [18], which did include acute WAD, there is to our knowledge no quantitative study presenting the outcomes of Internet-delivered treatment for neck pain, and the present study is the only one investigating chronic WAD, severe WAD, and exercise, for work-related factors in WAD.

The present study is a multicentre study involving treating physiotherapists in several units across a large geographical area, which is a risk due to having less control over treatments. However, the physiotherapists were well trained by the project team, they received carefully written, oral, and practical introductions and were free to contact the project leaders for support when needed. The advantage of a multicentre study may be that the study improved reproducibility, generalisability and potential clinical translation of the RCT. The participants themselves expressed their interest in participating in the RCT after seeing an advertisement for the RCT in various media. Prior to inclusion in the study, the criteria for participation were ensured, where the interviewer asked, among other things, whether the potential participant was prepared to carry out the program [22, 23]. This can lead to the results not being transferable to patients in ordinary healthcare and must be investigated in an implementation study without strict RCT criteria.

Individuals who were not able to read/write Swedish and thus unable to understand the information provided or answer the questionnaires were excluded, which makes it less generalizable to the whole population. However, in the future, the Internet-based programme can be translated into other languages, increasing the availability of this healthcare intervention for the whole population.

The power of the present article was counted on the main outcome in the RCT [22, 23]: the NDI, and not on work-related secondary outcomes. Nevertheless, the results of the main outcome and the work-related secondary outcomes support each other, with an improvement over time in both groups but without significant group differences. As clinically relevant improvement data was unavailable for the present outcomes and the chronic WAD population, non-inferiority analyses were not performed, as previously reported for the main outcome RCT data [22]. As previous RCTs conducted by our research team have shown three months of NSE to be cost-effective and superior to remaining on a waiting list or receiving prescribed general physical activity [7, 17, 40], we did not include a control group without specific treatment in the present RCT as it would have been unethical.

### Implications for Practice

In individuals with non-specific neck pain, neck strengthening exercises or increased physical activity have shown good results in work-related outcomes [43, 49]. In the present study, neck-specific exercise was tolerated with good results, without group differences and without adverse events [22], showing that NSEIT is as effective as NSE in improving and maintaining work ability for individuals with chronic WAD, but with fewer physiotherapy visits (four compared to 24 visits). As shown in our qualitative study, participating in this neck-specific exercise programme, including being acknowledged and receiving information about WAD, affected the individuals’ work ability [8]. The present study may help to provide evidence of treatment efficacy for patients with WAD and may help to optimize treatment plans for individuals with WAD. However, it is worth noticing that the results are presented at a group level and that NSEIT or NSE may not suit everyone, and for some individuals NSE may be preferred despite the large number of physiotherapy visits. Support for work participation for individuals with chronic WAD can be strengthened.

## Conclusion

One can conclude that, despite fewer physiotherapy visits, there were no group differences between NSEIT and NSE, with improvements in most work-related measures maintained at the 15-month follow-up. FABQ-work was close to significant in the NSEIT group at the 15-month follow-up. Thus, we accept the study hypothesis. The result of the present study may be promising for those with impaired work ability after a whiplash injury, although may not be generalisable to all individuals with WAD. More studies regarding rehabilitation for improved work ability are needed to optimize care, especially for those with chronic WAD. To achieve even higher effect sizes, NSE/NSEIT could be combined with rehabilitation, focusing on the work situation and factors improving work ability within a broad but individual context.

## Data Availability

Data contains information regarding health and will not be available, due to the Swedish Health Secrets Act, without specific ethical permission.

## Code Availability

IBM SPSS version 28 was used for statistical analyses.
